# Simultaneous Quantification of Anisotropic Microcirculation and Microstructure in Peripheral Nerve

**DOI:** 10.3390/jcm11113036

**Published:** 2022-05-27

**Authors:** Samer Merchant, Stewart Yeoh, Mark A. Mahan, Edward W. Hsu

**Affiliations:** 1 Department of Bioengineering, University of Utah, Salt Lake City, UT 84112, USA; samer.merchant@utah.edu; 2 Department of Neurosurgery, Clinical Neurosciences Center, University of Utah, Salt Lake City, UT 84132, USA; stewart.yeoh@utah.edu (S.Y.); mark.mahan@hsc.utah.edu (M.A.M.)

**Keywords:** DTI, IVIM, MRI, blood flow, perfusion, nerve, nervous system, injury

## Abstract

Peripheral nerve injury is a significant public health challenge, and perfusion in the nerve is a potential biomarker for assessing the injury severity and prognostic outlook. Here, we applied a novel formalism that combined intravoxel incoherent motion (IVIM) and diffusion tensor imaging (DTI) to simultaneously characterize anisotropic microcirculation and microstructure in the rat sciatic nerve. Comparison to postmortem measurements revealed that the in vivo IVIM-DTI signal contained a fast compartment (2.32 ± 0.04 × 10^−3^ mm^2^/s mean diffusivity, mean ± sem, *n* = 6, paired *t* test *p* < 0.01) that could be attributed to microcirculation in addition to a slower compartment that had similar mean diffusivity as the postmortem nerve (1.04 ± 0.01 vs. 0.96 ± 0.05 × 10^−3^ mm^2^/s, *p* > 0.05). Although further investigation and technical improvement are warranted, this preliminary study demonstrates both the feasibility and potential for applying the IVIM-DTI methodology to peripheral nerves for quantifying perfusion in the presence of anisotropic tissue microstructure.

## 1. Introduction

Peripheral nerve injury is involved in an estimated 5% of all trauma hospitalizations [1] and is a significant public health challenge that can lead to lifelong pain or loss of function [2]. Peripheral nerves have an innate capacity to regenerate in low-severity injuries; however, the determination of nerve injury severity is clinically opaque. The symptoms of nerve injury are variable, and appear in both mild injuries that recover and more severe injuries that require surgical intervention [3]. Unfortunately, only qualitative approaches are available for assessment of nerve injury severity or for prognostic outlook. Surgical treatment is thus predicated on failure to recover; which is established long past the optimal biological window for regeneration [4]. Although imaging techniques such as MRI have become standard practice for assessment of nerve anatomy, no technique yet exists that can reliably predict whether a nerve will recover after injury [5].

To support the metabolic needs of saltatory conduction along axons and glial cell populations such as Schwann cells, nerves are highly vascularized and are supplied by separate but interconnected intrinsic and extrinsic vascular networks [6,7]. Extrinsic vessels originate from nearby arteries and veins in adjacent tissues, whereas intrinsic vessels run longitudinally within the nerve. These linked and redundant systems are resilient to injury; however, sufficient disruption will result in ischemia [6], leading to progressive loss of conduction with worsening severity. Additionally, many nerve injuries such as positional palsies are primarily ischemic in character and may lead to long-term deficits. These factors make assessment of blood flow and perfusion in injured nerves an attractive target as an indicator of nerve health and regenerative capacity following injury.

MR imaging techniques such as multiplanar T1 and T2 and diffusion tensor imaging (DTI) have been used to evaluate microstructural injury and remodeling, but have had limited success in differentiating degenerating distal stumps from regenerating axons [5,8,9]. In particular, fractional anisotropy (FA) measurements from DTI have been observed to decrease in injured nerves [10,11,12] and have been correlated with successful nerve regeneration [9]; however, these studies are fundamentally retrospective and lack prognostic assessment.

MRI techniques sensitive to perfusion or blood flow are also available and include dynamic contrast imaging (DCE) [13], arterial spin labeling (ASL) [14], phase contrast [15], blood oxygenation level dependent (BOLD) [16], and intravoxel incoherent motion (IVIM) [17]. Recently, a formalism that combined IVIM and DTI, which we henceforth refer to as IVIM-DTI, was introduced and demonstrated in quantifying myocardial microcirculation [18]. This novel technique is uniquely suitable for simultaneously characterizing both anisotropic microstructure and partially coherent microcirculation expected in tissues such as nerves, without requiring the use of exogenous contrast agents. Although promising, the practical applicability of IVIM-DTI to peripheral nerves is currently unknown. For the above reasons, the goal of this first-of-its-kind study is to apply and validate IVIM-DTI to characterize peripheral nerve microcirculation in a preclinical rodent model commonly used in nerve injury research.

## 2. Materials and Methods

### 2.1. MRI

**Figure 1:**
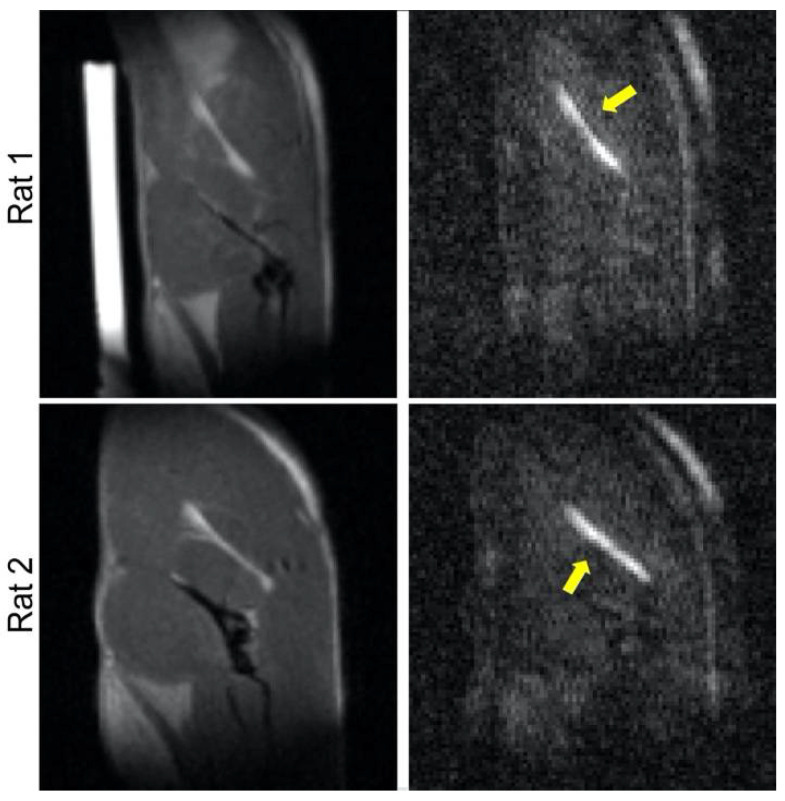
MRI of the rat sciatic nerve. Results are presented for 2 animals, with RARE and diffusion-weighted scans shown in the left and right columns, respectively. The latter are used to identify and confirm the locations of the nerve (arrows). The hyperintense cylindrical object in the top left image is a water-filled phantom used for reference purposes.

Using protocols approved by the University of Utah Institutional Animal Care and Use Committee, MRI was performed using a 7 T scanner (Bruker Biospin, Ettlingen, Germany) on the sciatic nerve in both right and left hind limbs of 3 female Sprague Dawley rats (Charles River, Wilmington, MA, USA). Briefly, the animals were anesthetized (3%) and maintained (~2%, adjusted when necessary) with isoflurane and placed in a lateral decubitus position with the top hind limb stretched and immobilized. A 2.0 cm-diameter surface coil (Bruker) placed above the thigh and a 72 mm volume coil (Bruker) were used as the radiofrequency (RF) receiver and transmitter, respectively. The sciatic nerve was first visualized (Figure 1) using anatomical sagittal-view, multi-slice T2-weighted RARE images (20 slices, 0.5 mm thickness, 4.0 × 3.6 cm FOV, 200 × 175 matrix size, 3 s TR, 34 ms TE, 4 averages and an echo train length of 8). Subsequently, using the same orientation and FOV, a multi-b-value DTI dataset consisting of diffusion weighted scans was acquired with a segmented echo planar imaging (EPI) sequence (8 segments, 10 slices, 0.5 mm slice thickness, 100 × 90 matrix size, 500 ms TR, and 19 ms TE) encoded in six ([1,0,0], [0,1,0], [0,0,1], [1,1,0], [1,−1,0], and [1,1,1]) directions with 8 b-values (nominally 25, 50, 75, 100, 200, 400, 600, and 1200 s/mm^2^). The combined scan time for the RARE and DTI datasets was approximately 35 min. To scan the second sciatic nerve, the animal was adjusted to be in the opposite lateral decubitus position with the second hind limb outstretched, and the entire imaging protocol was repeated.

After the in vivo scans, the animal was euthanized (100% CO_2_ at a fill rate of 30% of the chamber volume per minute) and the sciatic nerve in both thighs was rescanned using the IVIM-DTI protocol described above. Since no circulation was expected, these postmortem scans served as the control to validate the in vivo measurements. The postmortem scans were performed at an ambient temperature of 20 °C and completed within 75 min after euthanasia.

### 2.2. Data Analysis

All post-processing was done using custom codes written in MATLAB (R2019b, MathWorks, Natick, MA, USA). Postmortem DTI datasets were analyzed on a pixel-by-pixel basis using standard techniques [19,20] and scanner-provided, cross-term-corrected b-values to yield their diffusion tensors, denoted by $D_{post}$ (postmortem diffusion). In contrast, the in vivo DTI datasets were analyzed according to the previously introduced IVIM-DTI formalism, which modeled the diffusion signal essentially as a bi-exponential decay, consisting of fast and slow anisotropic diffusing compartments representing microcirculation ($D^{*}$) and microstructure ($D_{tissue})$, respectively, according to:

(1) $$s\left(b,\hat{g}\right)=s_{o}^{\prime }\exp\left(-b{\hat{g}}^{T}\cdot D_{tissue}\cdot \hat{g}\right)+s_{o}\exp\left(-b{\hat{g}}^{T}\cdot D^{*}\cdot \hat{g}\right),$$

where $\hat{g}$ is the encoding direction, and $s_{o}^{\prime }$ and $s_{o}$ are the unweighted (i.e., b=0) diffusion signal for the slow and fast compartments, respectively. To minimize fitting errors due to noise, a segmented approach [21,22,23] common in IVIM analysis was modified and used. This approach entailed (a) determination of the slow-diffusing compartments as tensor quantity $D_{tissue}\;$ (as opposed to conventional scalar diffusivity) and its intercept (i.e., $s_{o}^{\prime }$) using data only from images obtained with *b*-values greater than or equal to some $b_{cutoff}$ where the second term in Equation (1) was negligible, which then allowed for (b) conversion of the two-compartmental model to a more stable single-exponential numerical problem to find the second tensor quantity $D^{*}$, describing the faster-diffusing component along with its intercept $s_{o}$. For numerical fitting of all tensor quantities, the solutions were constrained to be symmetrically positive-definite, as described previously [20], to avoid negative eigenvalues.

A critical step in the above segmented fitting methodology was the determination of $b_{cutoff}$. Although most previous IVIM studies [22,24] of the brain used a $b_{cutoff}$ of 200 s/mm^2^, we did not assume the same could apply to peripheral nerves, since intuitively different degrees of microcirculation would necessitate a different threshold value. Instead, we visually inspected the semi-logarithmic plot of the mean signal intensity (over the sciatic nerve of each hind limb of each animal) of the in vivo diffusion images as a function of the *b*-value and empirically identified $b_{cutoff}$ from the knee or corner frequency in the slope of the plot, using the corresponding plot of the postmortem images as a reference for an approximately straight slope. Based on the median of the 6 cases examined (left and right nerves in 3 animals), we determined $b_{cutoff}$ for peripheral nerve microcirculation to be 400 s/mm^2^.

After finding $D_{post},\;D_{tissue}$, $D^{*}$, $s_{o}^{\prime }$, and $s_{o}$, the latter two scalar quantities were used to estimate the normalized vascular fraction f using

(2) $$f=\frac{s_{o}}{s_{o}+s_{o}^{\prime }}\;$$

The tensors then were diagonalized to yield their respective eigenvalues and eigenvectors. The eigenvalues, which represented the diffusivities along the principal axes, were in turn used to compute the fractional anisotropy (FA) and mean diffusivity (MD). For comparison, the parameters in question were averaged over the nerve region using a manually segmented ROI. The FA and MD values measured for nerve ROIs were tested using two-tailed paired t statistics, with *p* < 0.05 used as the criterion for significance between in vivo and postmortem scans.

## 3. Results

**Figure 2:**
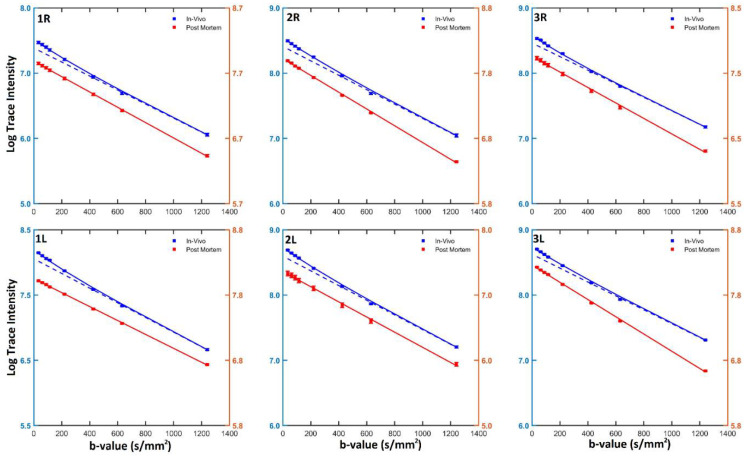
Diffusion signals in the rat sciatic nerve. The ROI-averaged trace-weighted diffusion signals obtained both in vivo (blue) and postmortem (red, with scales indicated to the right of the graphs) in the right (**top row**) and left (**bottom row**) sciatic nerves of all animals (**columns**) are plotted on a logarithmic scale as a function of the b-value. The solid lines denote the bi-exponential and single-exponential fits of the in vivo and postmortem data, respectively. The blue dashed lines are single-exponential fits of the in vivo data with *b*-value ≥ 400 s/mm^2^ and are extrapolated to lower b-values. Error bars represent standard errors of ROI mean.

Figure 2 shows the semi-logarithmic plots of the orientation-invariant diffusion tensor trace-weighted intensity as a function of the nominal diffusion-weighting b-value for all sciatic nerves observed in vivo and postmortem. The tensor trace-weighted intensity was obtained from the geometric mean of diffusion-weighted images encoded in the [1,0,0], [0,1,0], and [0,0,1] directions. Overall, the postmortem diffusion signal exhibited approximately straight-line (i.e., mono-exponential) decay, which justified the use of the same model for fitting the flow-independent diffusion compartment in the in vivo signal. In contrast, the in vivo signals had a small but noticeably steeper slope at a lower b-value range, which indicated the presence of an extra faster-diffusing compartment.

The median b-value where the change in the slope of the in vivo signal occurs among the six nerves examined was found to be 400 s/mm^2^, which was used as $b_{cutoff}$ in the two-compartmental analysis described above. The IVIM-DTI scalar quantities obtained from two-compartmental analysis of the in vivo diffusion signal for the individual nerves, including MD and FA derived from $D^{*}$ and $D_{tissue}$ (denoted by MD* and MD*_tissue_*, and FA* and FA*_tissue_*, respectively) and f, are tabulated in Table 1. Group-wise, MD*_tissue_* and the MD of $D_{post}$ (MD*_post_*) were 1.04 ± 0.01 × 10^−3^ mm^2^/s and 0.96 ± 0.05 × 10^−3^ mm^2^/s (mean ± sem, *n* = 6), respectively. Although MD*_post_* showed higher variability than MD*_tissue_* among individual animals and nerves, the difference between the two groups was not statistically significant (*p* = 0.175 by paired Student *t* test). The latter suggests not only that the choice of $b_{cutoff}$ was valid, but also that $D_{tissue}$ likely reflected diffusion in the absence of perfusion. The quantity of MD* was approximately twice as large as that of MD*_tissue_* (2.32 ± 0.04 vs 1.04 ± 0.01 × 10^−3^ mm^2^/s, *p* < 0.0001, paired t test), whereas FA* and FA*_tissue_* were comparable (0.50 ± 0.01 vs 0.53 ± 0.02, *p* = 0.259). These finding suggest that the microcirculation-induced diffusion was anisotropic (or partially coherent) within the voxel, and that the proportional increase in the observed diffusivity did not exhibit orientation preference. Lastly, the normalized vascular fraction of the nerve was found to be 0.15 ± 0.01.

**Figure 3:**
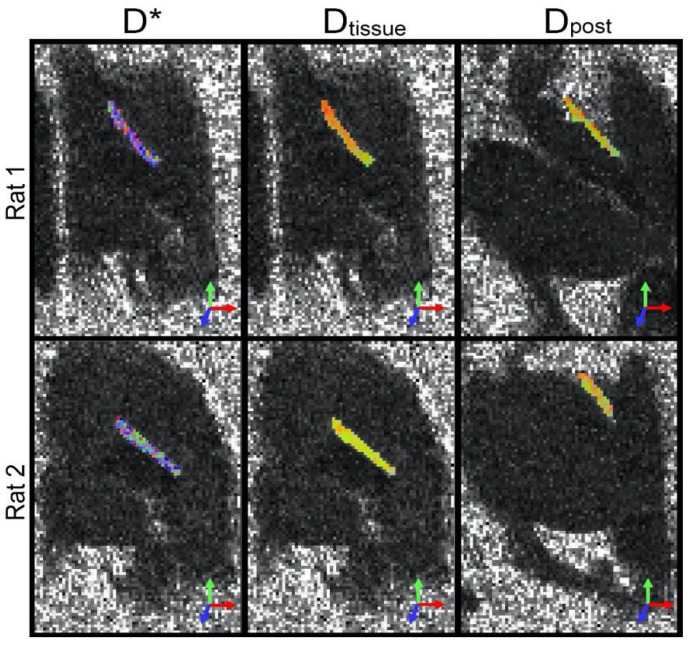
Diffusion orientations of the rat sciatic nerve. The primary eigenvectors of D* (i.e., fast compartment of the in vivo diffusion signal), **D***_tissue_* (slow compartment of the same), and **D***_post_* (postmortem diffusion signal) from two animals (separate rows) are RGB-coded, shown overlaid on their FA maps. The intensities of the images have been scaled to enhance contrast.

Figure 3 displays red–green–blue (RGB) false color-coded maps of orientations of the fastest diffusion (i.e., primary eigenvectors) of $D^{*}$, $D_{tissue}$, and $D_{post}$ of two different nerves overlaid on their respective FA maps. Overall, the primary orientation of anisotropy in $D_{tissue}$ and $D_{post}$ was not only relatively uniform, but also oriented along the axis of the nerve, further supporting the notion that the slow compartment in the in vivo diffusion signal is unrelated to microcirculation. In contrast, the orientation of the anisotropy of $D^{*}$ was rather heterogenous, indicating that microcirculation is incoherent macroscopically (between voxels).

## 4. Discussion

The central premise of the present study is that microcirculation in peripheral nerves induces a faster-decaying diffusion signal that can be distinguished from the slower-decaying signal associated with tissue microstructure such as axons, which also is seen with IVIM imaging of other tissues [18,25,26,27,28,29]. The results show that a faster-diffusing compartment clearly existed over a slower-diffusing compartment, at least in the normal rat sciatic nerve. Moreover, the slower in vivo diffusing compartment had similar MD as the postmortem nerve that lacked perfusion, which provides strong evidence to support the premise that the fast-decaying signal is associated with perfusion. The fact that both microcirculation- and microstructure-related compartments were highly anisotropic, with an FA of approximately 0.5 for both, underscores both the benefit and need to simultaneously but separately characterize their behavior using our IVIM-DTI formalism. We anticipate our method to be particularly advantageous for evaluating changes to microcirculation and tissue microstructure, which may not occur according to the established sequence in nerve injury and recovery, as blood vessels are known to respond to hypoxic conditions before Schwann cells and regenerating axons [30,31,32].

Clinical applications for a developed IVIM-DTI protocol are numerous, as many organ systems also have highly anisotropic vasculature and microstructure and would benefit from simultaneous but separate assessments of both. Nerves are a prototypical scenario where the microstructural integrity is reflective of the nerve health. However, in pathological conditions, DTI alone is a lagging indicator [33,34], and there is need to evaluate perfusion as a clinical biomarker for regeneration [35]. Further studies will need to evaluate the efficacy of IVIM-DTI in measuring microcirculation in healthy and pathophysiologic states of health. Furthermore, IVIM-DTI may benefit diagnosis of cardiac disease, where fibrosis and ischemia are both critical causes, and both of which have been independently examined using MRI techniques [36,37,38]. IVIM-DTI would potentially allow for the assessment of the perfusion and fibrotic state of specific regions of heart tissue. Since IVIM-DTI is diffusion MRI-based, one potential challenge for clinical adaptation is the availability of scanners with gradients powerful enough for the required spatial resolution and diffusion weighting. Although there is room for continued technical advancement, scanners equipped with 80 mT/m gradient sets capable of sub-millimeter resolution and b-values much greater than the 1200 s/mm^2^ used in the current study are already available.

As the first study to investigate differences in DTI signals from microcirculation, there are a few observations in the current study that warrant further investigation. First, the FA* reported in Table 1 suggests that the microcirculation-induced diffusion was highly anisotropic at the microscopic scale (i.e., within the voxel), whereas the RGB-coded orientation map in Figure 3 shows that the orientations of preferred diffusion lacked coherence macroscopically (or between neighboring voxels). One possible explanation for this discrepancy is that at the imaging resolution used (0.4 mm in-plane, and 0.5 mm slice thickness), any voxel will contain multiple capillaries that compromise the intrinsic intraneural blood supply and that generally point in the same orientation, which gives rise to the intravoxel anisotropy. However, at the intervoxel scale, larger vessels from the extrinsic supply and epineurium create disruption to the intervoxel coherence. The extrinsic supply arrives from larger vessels in surrounding tissue that approach the nerve at a perpendicular orientation and then split into numerous epineurial branches and anastomoses [7], which may account for local differences in voxel FA*. Note that the voxels at the imaging resolution used necessarily contained a high degree of volume averaging, leading to the overall FA alignment with the axis of the nerve.

The second observation of interest is that the MD* in Table 1 was relatively low compared not only to MD*_tissue_*, but also to the scalar D* reported for other tissues (typically in the range of 0.05–0.1 mm^2^/s [27]). Although the source of the low MD* is currently unclear, one immediate technical concern is that the difference between MD* and MD*_tissue_* may not have been large enough for accurate results to be obtained from the segmented two-compartment analysis, which is commonly thought to require an order of magnitude between the fast and slow diffusivities [29,39,40]. Intuitively, an insufficiently fast-decaying microcirculation-induced diffusion signal would lead to an overestimation of MD*_tissue_*. In the present study, MD*_tissue_* did appear to be slightly higher than MD*_post_*, but did not reach the threshold of statistical significance. These two quantities should arguably be the same, since microcirculation is in theory excluded in one and is absent in the other; however, the small decrease in MD*_post_* can be easily attributed to the lower temperature (20 °C instead of body temperature) at which it was measured. Consequently, the relatively small difference between MD* and MD*_tissue_* is unlikely to have compromised our segmented two-compartmental analysis.

Lastly, other areas that require additional inquiry include the behavior of the diffusion signal and the FA of $D_{tissue}$ and $D_{post}$ (i.e., FA*_tissue_* and FA*_post_*). For example, the source of the variability observed in MD*_post_* is currently unclear, albeit possible causes include postmortem temperature and water compartment changes. It is also unclear why some postmortem diffusion signals in Figure 2 exhibited a slight curvature. Moreover, whereas MD*_tissue_* and MD*_post_* were comparable, as shown in Table 1, FA*_post_* was significantly lower than FA*_tissue_* (0.38 ± 0.02 vs. 0.53 ± 0.02, *p* < 0.001). On one hand, the difference could be a technical artifact caused by fitting $D_{tissue}$ from images acquired with only three b-values, and that noisier DTI data could lead to inflated FA values [41,42]. On the other hand, the dissimilar values could point to real changes in the FA associated with microstructural changes upon animal death, i.e., rigor mortis. In support of this theory is that the FA*_post_* of the right sciatic nerves, which coincidentally were imaged first in all animals, were larger than those for the left, albeit the sample size is too small to be conclusive. In hindsight, to clarify current findings or to extend the current study, future investigations can benefit from better techniques (e.g., more b-values in fitting $D_{tissue}$ and higher spatial-resolution scans) and design (using a transient tourniquet as a control). However, we do not believe the need for improvements detracts from the significance and potential of the current study.

## 5. Conclusions

In summary, we applied a novel methodology that combined IVIM and DTI capable of simultaneously assessing microcirculation and microstructure to image the rat sciatic nerve both in vivo and postmortem. Two-compartmental analysis and comparison to postmortem measurements revealed that microcirculation induced an additional and faster compartment in the in vivo IVIM-DTI signal. Although the present preliminary study only demonstrated its feasibility and validity in normal nerves, we anticipate that the proposed methodology will become an invaluable clinical tool for evaluating the severity of nerve injury and regenerative potential.

## Figures and Tables

**Table 1:** Tensor-derived quantities of the in vivo and postmortem diffusion signals, with the in vivo signal separated into fast and slow components.

Rat	Limb	Fast Component		Slow Component		Vascular Fraction	Postmortem	
		MD* (mm^2^/s)	FA*	MD*_tissue_* (mm^2^/s)	FA*_tissue_*	f	MD*_post_* (mm^2^/s)	FA*_post_*
**1**	**Right**	2.38	0.50	1.08	0.55	0.15	0.76	0.41
	**Left**	2.35	0.49	1.02	0.48	0.16	0.84	0.31
**2**	**Right**	2.36	0.51	1.07	0.60	0.16	1.04	0.42
	**Left**	2.24	0.51	1.01	0.50	0.18	1.01	0.37
**3**	**Right**	2.46	0.48	1.02	0.56	0.15	1.05	0.46
	**Left**	2.13	0.51	1.06	0.49	0.12	1.04	0.35
**Mean**		**2.32**	**0.50**	**1.04**	**0.53**	**0.15**	**0.96**	**0.38**
**±sem**		0.04	0.01	0.01	0.02	0.01	0.05	0.02

## Data Availability

Study data available on request.
